# Molecular Mechanisms of Preeclampsia

**DOI:** 10.1155/2012/298343

**Published:** 2012-02-23

**Authors:** N. Vitoratos, D. Hassiakos, C. Iavazzo

**Affiliations:** 2nd Department of Obstetrics and Gynecology, University of Athens, Aretaieion Hospital, 11528 Athens, Greece

## Abstract

Preeclampsia is one of the leading causes of maternal morbidity/mortality. The pathogenesis of preeclampsia is still under investigation. The aim of this paper is to present the molecular mechanisms implicating in the pathway leading to preeclampsia.

## 1. Introduction

 Preeclampsia is one of the leading causes of maternal morbidity/mortality and preterm delivery worldwide [1]. It is a syndrome defined by the onset of hypertension (≥140/≥90 mmHg) and proteinuria (≥0,3 gr/24 h) after 20 weeks of gestation in a previously normotensive woman that also may be associated with myriad, other signs and symptoms, and often with subnormal fetal growth [2, 3]. Most commonly, preeclampsia occurs in healthy nulliparous women. However, multiparous pregnant women with a new partner have an increased risk of preeclampsia similar to that of nulliparous women [4]. Women with a history of preeclampsia in a prior pregnancy are at increased risk of developing preeclampsia in future pregnancies [5]. A history of preeclampsia in the father's mother also confers an increased risk [6]. Several medical conditions, such as chronic hypertension, diabetes mellitus, renal disease, and hypercoagulable states are associated with increased preeclampsia risk [7, 8]. Additionally, obstetrical conditions with increased placental mass increase the risk of preeclampsia. These include hydatidiform mole [9] and multifetal gestation [10].

 Delivery of the placenta remains the only known treatment for this clinical disease, suggesting that the placenta is the principal contributor to the pathogenesis of preeclampsia. It is well known that the first step for the development of preeclampsia is the inadequate placental cytotrophoblast invasion, impaired trophoblast invasion, and inadequate maternal spiral artery remodeling which results in placental ischemia and hypoxia. However, placental ischemia does not always generate the clinical symptoms of preeclampsia. Many molecular mechanisms are contributed to the pathogenesis of preeclampsia. Altered angiogenic balance, systemic inflammation, dystregulation of renin-angiotensin system, and placental hypoxia and ischemia are mechanisms which contribute to the pathogenesis of pre-eclampsia, although it is unknown whether the mechanisms act independently or have synergistic effects.

## 2. Altered Angiogenic Balance

### 2.1. Angiogenic Factors

 A variety of angiogenic factors are produced from the human placenta. The most important between them are the vascular endothelial growth factor (VEGF) and the placental growth factor (PIGF) [11]. VEGF is an endothelial-specific mitogen that plays a key role in promoting angiogenesis. VEGF stabilizes endothelium in mature blood vessels [12]. VEGF'S activities are mediated primarily by its interaction with two high-affinity receptors tyrosine kinases—kinase-insert domain region (KDR or VEGF R-2) and fms-like tyrosine kinase-1 or flt-1. Both receptors are expressed on vascular endothelial cell surface [13]. PIGF is also an angiogenic growth factor that is thought to amplify VEGF signalling by displacing VEGF from the flt-1 receptor and allowing it to bind to the more active kinase-insert domain (KDT) receptor [14, 15].

 Recent research has shown that soluble flt-1 is released by the placenta into the maternal circulation and, that is, contributes to the hypertension, proteinuria, and endothelial cell dysfunction associated with preeclampsia [16]. sflt-1 antagonizes both VEGF and PIGF by binding them in the circulation and preventing interaction with their endogenous receptors [17, 18]. New variants of sflt-1 have been discovered such as sflt1–14, which is also a potent VEGF inhibitor [19, 20]. The level of SFLt-1 in the plasma of women with preeclampsia is elevated and that of VEGF is diminished in comparison with that of women with complicated pregnancies [21]. Furthermore, administration of sflt-1 to rats resulted in elevated blood pressure and proteinuria, indicating that excessive placenta-derived sflt-1 max contributes to preeclampsia [21].

 Factors responsible for excessive production of self-1 in preeclampsia have not been identified. However, recently it has been found that angiotensin II type 1 (AT) receptor autoantibodies which occur in women with preeclampsia contribute to increased production of sflt-1. Thus, IgG from women with preeclampsia stimulates the synthesis and secretion of sflt-1, via AT_1_ receptor activation in human placental villous explants and human trophoblast cells. Another factor which contributes to increased production of sflt-1 is the hypoxic placenta [22]. Under other pathophysiological conditions such as cancer and hypoxia which generally stimulates angiogenic signalling, it remains poorly understood why hypoxic placenta produces the molecules that suppress angiogenesis in preeclampsia [23].

 Soluble endoglin is another antiangiogenic protein, which acts to get it with sflt-1 to induce a severe preeclampsia-like syndrome in pregnant rats. Circulating soluble endoglin levels increased markedly beginning from 2 to 3 months before the onset of preeclampsia. An increased level of soluble endoglin was usually accompanied by an increased ratio of sflt-1 [24].

 Experiments data have shown that VEGF stimulates the production of both nitric oxide (NO) and PGI_2_ [25]. On the other hand, a high concentration of asymmetric dimethylarginine (ADMA), an endogenous inhibitor of endothelial nitric oxide synthase, has been found in preeclamptic women [26].

 Women with bilateral notches who later developed preeclampsia had a striking elevation in the concentration of the NO synthase inhibitor [27].

 ADMA is normally metabolized to citrulise through the action of dimethylarginine-dimethylaminohydrolase I, II (DDAH I, II). Oxidative stress seen in preeclampsia diminishes the action of the alone enzymes leading to high concentration of ADMA [27].

## 3. The Role of Relaxin in Preeclampsia

 Relaxin is produced by the corpus luteus of the ovary and rises early in pregnancy, and chorionic gonadotropin produced by the placenta is a major stimulus for relaxin secretion during pregnancy.

 Relaxin has renal vasodilatory effect [28], and it also diminishes the relaxin vasoconstrictor response to angiotensin II. Moreover, reduced myogenic reactivity of small renal arteries is observed after relaxin administration [29, 30]. Recently, it has been proposed that relaxin via relaxin receptor upregulates vascular gelatinase activity during pregnancy, contributing to renal vasodilation through activation of endothelial endothelin B (ET_B_) receptor which activates nitric oxide synthase III and the production of NO [31]. Thus, increased vascular gelatinase activity by relaxin is thought to be a proximal step in the vasodilatory pathway of pregnancy [32].

 Circulating levels of immunoreactive relaxin have been reported to be similar in women with preeclampsia and normal pregnancy [33]. However, whether circulating relaxin bioactivity may be deficient during the disease is uncertain [34]. Furthermore, mutations or polymorphisms of the ET_B_ receptor or of endothelial NO synthase that reduce activity may predispose a woman to preeclampsia by impairing trophoblast invasion on the one hand or by compromising maternal endothelial behaviour on the other [35, 36].

## 4. Inflammatory Cytokines in the Pathophysiology of Preeclampsia

 Reduced uterine perfusion during pregnancy is an important initialling event in preeclampsia. Inflammatory cytokines are thought to link placental ischemia with cardiovascular and renal dysfunction [37]. In normal pregnancy TNF-*α* is low in the first trimester and subsequently increases with advancing gestation age [38]. Some studies report higher TNF-*α* levels in women with established preeclampsia [39, 40]. Increased levels of TNF-*α* antigen and mRNA have been described in placental tissue from preeclamptic women [41].

 Because TNF-*α* may impair insulin signalling, inhibit lipoprotein lipase, induce PAI-1, and directly contribute to endothelial dysfunction, this cytokine may be involved in the pathogenesis of preeclampsia [42].

 There are also findings showing that chronic infusion of IL-6 into normal pregnant rats stimulates the renin-angiotensin system (RAS) [37].

 Natural killer (NK) cells, dendritic cells, and macrophages are mediators of innate immunity. Macrophages and dendritic cells are the major antigen-presenting cells in the uterus, and they facilitate adaptation of the immune response to prevent rejection of the embryo [43].

 Several studies have found a statistically significant increase in macrophages and dendritic cells in preeclamptic placentas compared to placentas from normotensive pregnancies [43, 44]. An increase in the concentration of cytokines, molecules capable of recruiting macrophages, and dentritic cells has also been found in preeclamptic placentas [44].

 The increased presence of cytokines, macrophages, and dendritic cells in preeclamptic placentas supports the hypothesis that an inflammatory milieu presents in women with preeclampsia [44].

## 5. Activation of Renin-Angiotensin System (RAS)

 Renin-angiotensin system is one that controls blood pressure [45]. The expression of rennin mRNA was detected in human deciduas, macrophages, chorioamniotic membrane, and vascular smooth muscle cells [46].

 Angiotensin II receptor type I (AT_1_) was shown to be localized both in villous and extravillus trophoblasts, and this AT_1_ responds to exogenously administrated angiotensin II [47].

 The circulating level of angiotensin II increases as the pregnancy advances [48]. In preeclampsia, the circulating level of angiotensin is rather decreased [49], despite the fact that the vascular sensitivity to angiotensin is elevated in hypertensive pregnant women.

 The AT_1_ receptor gene expression was higher in placenta than in deciduas for both normal and preeclamptic women. However, the deciduas of preeclamptic women has a significantly higher AT_1_ receptor gene expression than normal pregnant women [50]. It has been found that the gene encoding the AT_1_ receptor was upregulated in the deciduas of preeclamptic women but not in normal control [51]. Circulating agonistic autoantibodies directed at the angiotensin II type 1 receptor (AT_1_-AA_s_) have been discovered in women with preeclampsia [52].

 Thus the increased decidual AT_1_ expression in pre-eclampsia may be the initial step for a profound RAS activation. Furthermore, the presence of AT_1_-AA_s_ is able to activate cells via the AT_1_ receptor and initiate signaling events that could contribute to development of preeclampsia. Thus, release of soluble flt-1 can be triggered by angiotensin II stimulation, raising an imbalance between angiogenic vascular endothelial growth factors and antiangiogenic soluble factors [21]. Zhou et al. [21] have shown that the inhibition of AT_1_ administration of losartan or FK506 resulted in reduced SVEGFR-1. Thus, maternal SVEGFR-1 can be elevated not only by poor placentation but also by AT_1_ activation in which angiotensin II and AT_1_-AA_s_ are potentially implicated.

## 6. Placental Hypoxia and Ischemia

 Impaired trophoblast invasion and inadequate maternal spiral artery remodelling result in placental ischemia and hypoxia. It is unknown, however, whether abnormal placentation leads to systemic vascular dysfunction and the appearance of preeclampsia.

 Defective trophoblast invasion and inadequate maternal spiral remodelling frequently result in intrauterine growth restriction or other complications of pregnancy (preterm labor e.g.) without preeclampsia even to normal full-term pregnancy [53]. Women living in high altitudes have an increased risk of developing preeclampsia [54], while cigarette smoking is associated with a reduced risk for preeclampsia [55]. Experiments in animals suggest that placental hypoxia contributes to preeclampsia by upregulating soluble antiangiogenic factors, inflammatory cytokines, downregulating angiogenic, and vasodilator factors [56].

 Furthermore, in pregnant mice, an absence of 2-methoxyestradiol (2-ME), a natural metabolite of estradiol, results in a deficient of catechol-o-methyltransferase (COMT). These animals showed a preeclampsia-like phenotype [57]. The addition of 2-ME was shown to improve preeclampsia and suppress placental hypoxia and sflt-1 expression [57]. It is, however, unclear whether or not decreased COMT is the cause of the consequence of impaired placentation.
